# Bioactive Materials in Pediatric Endodontics: Current Applications and Future Directions

**DOI:** 10.7759/cureus.90718

**Published:** 2025-08-22

**Authors:** Abdulrahman S Alshalan, Fai A Almutiri, Ali H Al-battat, Abdulrahman M Alqahtani, Khalid A Binzamil, Reem M Alabdan, Khalidah K Alrabghi, Asma M Aldohailan, Eman A Alshammari, Abdulrahman S Khurayniq, Mazen T Alshahrani

**Affiliations:** 1 Pediatric Dentistry, King Saud Medical City, Riyadh, SAU; 2 Dentistry, King Saud Bin Abdulaziz University for Health Sciences, Riyadh, SAU; 3 Dentistry, Ram Clinics, Al-Ahsa, SAU; 4 Dentistry, Security Forces Hospital, Dammam, SAU; 5 Dentistry, King Saud University, Riyadh, SAU; 6 Dentistry, Armed Forces Hospital Al-Qassim, Buraydah, SAU; 7 Dentistry, Ministry of Health, Taif, SAU; 8 Dentistry, Imam Abdulrahman Bin Faisal University, Alkhobar, SAU; 9 Dentistry, University of Hail, Hail, SAU; 10 Dentistry, Taibah University, Madinah al-Munawwarah, SAU; 11 Dentistry, Khamis Mushayt Maternity and Children's Hospital, Khamis Mushayt, SAU

**Keywords:** bioactive materials, biocompatibility, pediatric endodontics, pediatric pulp therapy, tissue regeneration

## Abstract

Bioactive materials have considerably advanced the field of pediatric endodontics by facilitating treatments that foster tissue healing, regeneration, and the long-term preservation of natural teeth in children. The principal objective of pediatric endodontic care is to sustain the functionality and health of primary teeth until their natural exfoliation occurs. The latest generations of materials employed in these treatments are engineered to interact positively with dental tissues, thereby promoting biological responses and repair processes. These innovations enhance sealing properties, minimize procedural complications, and support the development of natural tissues. While there are practical limitations, such as cost and restricted access in certain regions, ongoing advancements continue to enhance the clinical applicability of these materials. As research progresses, bioactive materials are becoming increasingly essential to minimally invasive, biologically oriented pediatric dental care, which supports both oral health and overall developmental and growth.

## Introduction and background

Pediatric endodontics is a critical branch of dentistry that deals with the management of root canal therapy in primary teeth to preserve oral health in children [1]. The main aim is to keep primary teeth functional up to their natural exfoliation, as they are essential for correct speech, nutrition, and permanent tooth alignment [2]. The major pulp therapy procedures in pediatric endodontics are indirect pulp therapy, pulpotomy, pulpectomy, and root canal fillings, each of which is specific to the tooth condition and root development stage [2]. One basic principle is pulp vitality maintenance in immature permanent teeth for further root development and apexogenesis [2]. More sophisticated instruments, such as electronic apex locators and rotary nickel-titanium files, are increasingly used to ensure optimum treatment accuracy and efficiency. Accurate preoperative diagnosis and assessment are essential for success. Treatments must also be adapted to the stage and age of the developing child so that they are compatible with the dynamic characteristics of pediatric dentition [1-8].

Bioactivity, in simple terms, refers to the ability of a dental material to actively interact with surrounding tissues rather than merely serving as a passive filler. Unlike traditional inert materials that only restore form and function, bioactive materials can stimulate healing, promote dentin formation, and bond chemically with tooth structures [7-8]. It is important to note that most of these bioactive materials were first developed and widely used in adult endodontics; however, their application in children requires careful adaptation due to factors such as smaller tooth dimensions, shorter chair time, and the dynamic growth patterns of pediatric patients [7-8].

Pediatric endodontics is increasingly focusing on the use of biologically active materials to facilitate treatment, maintain tooth vitality, and meet the specific requirements of growing children. These materials have been found to promote the treatment of pulp and periapical tissues, encourage dentin formation, and support the ongoing development of roots in the teeth with open apices [3]. Calcium is known for its outstanding compatibility with bioactive materials, biological tissues, and their active properties, such as silicate-based bioceramics like Mineral Trioxide Aggregate (MTA) (ProRoot MTA - Dentsply Sirona, Charlotte, USA) and Biodentine (Septodont, Saint-Maur-des-Fossés, France). They can create chemical bonds with tissues and positively affect stem cells, osteoblasts, and fibroblasts [4]. This makes bioactive materials especially useful in pediatric endodontics, where pulp vitality has to be maintained and continuous dental growth encouraged. Endodontic management of open apices teeth poses special problems in terms of promoting root development and pulp health [4]. In such cases, materials such as Biodentine have gained popularity because of their positive physical and chemical characteristics, compatibility with biological systems, and potential to stimulate dentin formation. In essence, not only does the use of bioactive materials solve existing problems, but it also contributes to future health and stability [3-4].

This article is presented as a narrative review, aiming to synthesize and critically discuss current evidence on bioactive materials in pediatric endodontics. Sources were identified through searches of PubMed and Google Scholar, focusing on recent clinical studies, case series, reviews, and in vitro research relevant to pediatric endodontic practice. High‑quality evidence, such as randomized controlled trials and systematic reviews, is highlighted where available.

## Review

Concept and evolution of bioactivity in endodontics

In endodontics, bioactivity refers to the ability of materials to act in a positive manner with the surrounding tissue, stimulating healing and tissue regeneration. This concept has progressed over recent years with increased focus on materials that effectively provide mechanical strength while engaging in biological repair mechanisms. Bioactive materials are defined as materials that create certain chemical bonds with surrounding tissue in order to induce regeneration [5]. They are utilized far more today, especially in regenerative endodontic treatments (RETs), where they serve as cell adhesion, proliferation, and differentiation scaffolds [6]. These include materials such as calcium silicate-based cements that possess ion-releasing capability to stimulate healing. The extent of bioactivity is determined by hydrogel concentration, the solubility of the incorporated bioactive molecules, and the apical dimension of the treated tooth [6]. Recent studies aim to optimize such parameters and create mathematical models that can forecast the clinical performance of bioactive materials [6]. Further, advanced technologies such as artificial intelligence are being investigated to create individualized endodontic hydrogels based on individual patient profiles. Such developments in the field of material science indicate a shift from passive to biologically interactive materials for enhanced treatment success and long-term oral health [5-6].

It is important to note that most bioactive materials were initially developed for adult endodontics; however, their clinical application in children requires careful adaptation. Factors such as the smaller dimensions of primary and immature permanent teeth, the need for shorter chair time, and the dynamic growth patterns of pediatric patients necessitate modifications in handling, setting time, and delivery of these materials to ensure optimal outcomes [7-8].

Bioactive endodontic materials are chemicals that can trigger specific chemical reactions and induce an optimal biological response at the intersection of the dental tissue and the material itself [5]. In contrast to traditional inert dental materials, bioactive materials are unique in that they promote the healing and regeneration of periradicular and pulpal tissues [7]. One basic property of bioactive materials is their ability to create a chemical bond with surrounding dental structures, thus increasing the stability and duration of restorative results [5]. In addition, these materials positively stimulate the regeneration of the surrounding tissues, leading to recovery of function and tissue repair [7]. They are most typically made up of calcium silicate-based materials, which are highly biocompatible and release calcium ions that contribute to mineralization and healing [7]. Their use covers a broad array of endodontic treatments ranging from pulp capping, pulpotomy, and root perforation repair to retrograde fillings in apical surgeries [7]. They benefit both the patient and the clinician, as they are not just more beneficial for the treatment outcomes but also easier to handle, providing clinicians with handling benefits and clinical efficiency. As a result of their biological efficacy and multifunctionality, bioactive materials have become unavoidable in modern endodontics [5,7].

Bioactive materials have revolutionized pediatric endodontics by improving treatment results and promoting biologically desirable characteristics for healing and regeneration of dental tissues. Their use in pediatric endodontics started in the 1990s and represented a major advancement in endodontic material science [7]. The advent of MTA was a breakthrough; it showed superior sealing quality, biocompatibility, and the ability to produce hard tissue formation, and it was thus indicated for several pediatric procedures [8]. MTA, however, had shortcomings like tooth darkening and extended setting time. Therefore, other substances like Biodentine, BioAggregate (Innovative BioCeramix Inc., Vancouver, Canada), and Calcium-Enriched Mixture (CEM) (BioniqueDent, Tehran, Iran) were developed to overcome these disadvantages while maintaining bioactivity [8]. These materials have since been used in critical pediatric endodontic treatments such as direct and indirect pulp capping, pulpotomy, repair of perforations, apexogenesis, and apexification [8]. The scientific interest garnered by these materials is clear from the availability of over 2500 articles and 200 reviews on bioactive tri/dicalcium silicate dental materials, highlighting their clinical significance and increasing influence [7]. Currently, bioactive materials are becoming a pillar in pediatric dentistry because they exhibit higher biocompatibility, lower cytotoxicity, and the potential to induce dentinogenesis, effectively promoting the conservation and growth of juvenile dentition [7]. 

In pediatric dentistry, bioactive materials are widely applied in procedures such as direct and indirect pulp capping, pulpotomy, apexogenesis, and apexification. Their ability to promote dentin bridge formation and continued root development makes them especially valuable in treating immature permanent teeth and preserving pulp vitality in young patients [8-36].

Biomaterials employed in pediatric tissue engineering have advanced greatly, with emphasis on inducing tissue repair, regeneration, and long-term function attuned to the dynamic growth patterns of children [4]. The optimal biomaterial should be biocompatible, malleable, and conducive to developmental alterations over time. Among the most important innovations, synthetic resorbable materials have exhibited significant safety, with a low infection rate of 1.1% in a large analysis involving 3,573 pediatric cases [9]. In contrast, natural biomaterials, although biologically optimal, exhibited a slightly increased infection rate of 5% based on the analysis of 298 pediatric cases [9]. Multifunctional composite hydrogels are a major development in which nanomaterials, drugs, stem cells, and bioactive molecules are combined to improve antibacterial activities, hemostasis, and regeneration properties. They create a beneficial healing environment and enable clinical improvement in tissue repair. Biomaterials coupled with drug delivery systems allow for the controlled and sustained release of therapeutic drugs like antibiotics and growth factors, reducing side effects while promoting efficient wound healing. These developments aim to design biologically active, functional scaffolds that are alternatives to conventional treatments, offering safer and more efficient solutions for pediatric surgery procedures and tissue engineering applications [4,9-10].

Types of bioactive materials in current use

The following table summarizes the main types of bioactive materials currently used in pediatric endodontics. It highlights their key advantages and disadvantages alongside relevant references. Each material offers unique benefits for dental healing and regeneration, but also has specific limitations that may affect its clinical use. The table provides a quick comparison to help guide material selection in practice (Table 1).

**Table 1:** Advantages and Disadvantages of Each Material MTA: mineral trioxide aggregate, CEM: calcium-enriched mixture.

Material	Advantages	Disadvantages	Citation
MTA	High biocompatibility, excellent sealing, promotes tissue regeneration and healing, wide clinical use	Long setting time, high cost, potential for tooth discoloration	[11-12]
Biodentine	Quick setting, easy handling, less discoloration, promotes dentin regeneration, high biocompatibility	Still needs more long-term studies, may not always match MTA in all indications	[13-15]
CEM Cement	Similar to MTA in effectiveness, quick setting, good handling, supports tissue regeneration, and high osseoconductivity	Limited long-term data, less research compared to MTA	[16-19]
Bioceramic Sealers	Excellent sealing, strong dentin adhesion, highly biocompatible, supports bone healing, fast healing	Newer in the market, long-term performance is still under review	[20-22]
Nanoparticle-Reinforced Materials	Enhanced antibacterial action, deeper penetration, supports tissue regeneration, multifunctional	Long-term safety and toxicity concerns, more clinical studies are needed	[23-26]

While bioactive materials offer clear benefits for pediatric endodontics, a critical evaluation of the existing literature is needed. Many published studies are limited by small sample sizes and short follow-up periods, making it difficult to draw firm conclusions about long-term effectiveness. Conflicting outcomes between different materials are reported, particularly when comparing MTA and Biodentine for pulpotomy and apexification procedures. Some studies favor MTA for long-term success, while others find Biodentine equally effective in the short term. However, some high‑quality studies, including randomized controlled trials and systematic reviews, have been published, and these often highlight the need for longer follow‑up periods and larger, more diverse patient groups. Therefore, clinicians should interpret reported success rates with caution and consider the methodological limitations when selecting materials for pediatric endodontic patients [20‑42].

MTA

MTA, a first-generation bioactive material with superior physical and biological properties, has been widely used in most endodontic treatments [11-43]. Due to its high sealing ability, low solubility, and intense bioactivity, it has several uses, such as in vital pulp treatment, perforation repair, apexification, root-end fillings, and regenerative endodontics [11]. Its biocompatibility is among the main characteristics of MTA that enable it to interact with periapical tissues in a favorable manner. Upon contact with tissue fluids, MTA facilitates hydroxyapatite crystal growth on the material-tissue interface, which stimulates the development of a calcified barrier necessary for regeneration and healing [11]. Notwithstanding these fundamental benefits, MTA has limitations as well. The material has a relatively long setting time, making procedures in the clinical setting more challenging. It is also linked with high costs and tooth discoloration potential, especially in aesthetic-sensitive areas, limiting its application in specific clinical conditions [11]. Before the introduction of newer MTA-based materials, clinicians attempted to manage these drawbacks using interim strategies. Discoloration was sometimes mitigated by placing a protective liner, such as resin-modified glass ionomer, over MTA in esthetic areas. To address the long setting time, practitioners often adopted a two-visit approach, placing a moist cotton pellet and completing the restoration at a subsequent appointment. Additionally, in cases where cost or handling was prohibitive, calcium hydroxide and other traditional pulp-capping agents were employed despite their inferior sealing ability and reduced long-term success [12-14].

To overcome these challenges, newer MTA-based materials have been designed. These have tried to preserve the desirable properties of the original material while improving handling characteristics and limiting disadvantages like discoloration and delayed setting time, thus widening its scope in contemporary endodontics [11-12]. Several newer bioactive calcium silicate‑based cements have recently emerged, for example, NeoMTA Plus (Avalon Biomed, Brasseler, USA) and NeoPUTTY (Avalon Biomed, Brasseler, USA), which demonstrated exceptionally high clinical and radiographic success rates (100% clinical, ~97 % radiographic at 12 months) in randomized trials of primary molar pulpotomies. These materials also show reduced tooth discoloration, high cytocompatibility, and favorable calcium phosphate deposition supporting healing [43].

MTA has been of significant interest in pediatric endodontics due to its favorable physical and biological characteristics and its applicability in a wide variety of pediatric patients' procedures [11]. It is strongly recommended for vital pulp procedures such as direct pulp capping and pulpotomy, in which biocompatibility and sealing ability contribute to successful clinical outcomes [11-12]. In necrotic pulp conditions with open apices, as routinely seen in immature permanent teeth, MTA is used successfully for apexification to form a consistent apical barrier [11-12]. MTA is also very important in regenerative endodontics as a shielding scaffold above the blood clot or biomaterial matrix, facilitating the regeneration of the tissue in immature teeth [12]. Its capacity to repair perforations makes it perfect for cases of iatrogenic or pathological perforations of pediatric teeth [11-12]. One important characteristic of MTA is its bioactivity, as it induces the formation of hydroxyapatite crystals upon contact with tissue fluids, stimulating calcific barrier formation and improved healing outcomes [11]. Although it has clear advantages, continued clinical studies are needed to determine its long-term effectiveness and compare it with newer agents in pediatric endodontics [11].

Biodentine

Biodentine, a new calcium silicate-based material, has been developed as an emerging alternative to the conventional materials such as MTA, primarily in pediatric endodontics, owing to its advantageous physical and biological characteristics [13]. It possesses several benefits over MTA in terms of quicker setting times, reduced risk of tooth discoloration, and better handling properties through its finer particle size [13]. Biodentine has been found to enhance dentin regeneration and pulp vitality, which are of utmost significance in the treatment of developing permanent and primary teeth [14]. It is highly versatile and is used in the treatment of direct pulp capping, pulpotomy, root perforation repair, apexification, and retrograde filling [15]. Furthermore, clinical trials have been favorable for Biodentine success rates in permanent mature teeth with irreversible pulpitis, with success rates of 98.4% at six months and 100% at twelve months [15]. Its biocompatibility has been established in research where there was only a mild or nonsignificant inflammatory response within 14 days of placement [14]. These aspects make it especially indicated among pediatric patients, where tissue response and regeneration are critical. While its benefits are clear, further research and long-term clinical trials are needed to confirm its effectiveness and credibility in pediatric endodontic procedures [15].

CEM Cement

CEM cement has shown vast potential as a bioactive cement in pediatric endodontics, and it is effective in primary and immature permanent teeth. In vital pulp treatment, CEM cement has also shown positive results in primary molar pulpotomies, with outcomes equal to MTA [16]. It also facilitates apexogenesis, as complete apical closure in 76.8% of immature permanent molar roots occurred within 12 months, which is comparable to MTA at 73.8% [17]. The material encourages the formation of dentinal bridges, causing thick tubular dentin bridges at pulpotomy sites [16]. In addition, it has been found to facilitate complete bridge formation without resulting in pulp canal obliteration within a five-year period of time. CEM cement also exhibits good biocompatibility and osseoconductivity, essential for pediatric use [18]. As it has better handling properties and significantly reduced setting time than MTA, it becomes extremely beneficial in children [18]. Its regenerative application is further validated by its capacity to facilitate dentinogenesis and cementogenesis, essential for forming teeth [19]. Moreover, it is versatile, with various applications, including open apices and root resorption repair [19]. It develops higher surface microhardness over time, which reflects improved long-term stability. All these characteristics make CEM cement an appropriate and strong material for pediatric endodontics [18-19].

Bioceramic Sealers

Bioceramic sealers are gaining prominence as potent materials in pediatric endodontics due to their favorable characteristics. They are excellent sealers of the root canal system, which is essential for successful treatment [20-21]. They are highly biocompatible even when used at high concentrations and therefore are perfectly suited to be applied in pediatric cases [20]. They facilitate osteoblastic differentiation, thus aiding bone healing [22], and exhibit strong adhesion to dentin, which increases treatment longevity [22]. Clinical trials indicate excellent success rates using EndoSequence BC Sealer (Brasseler USA, Savannah, USA) (94.9%) and NeoSealer Flo (Avalon Biomed Inc., Bradenton, USA) (96.5%). Moreover, they promote quick healing of tissue and are efficient in both primary and retreatment scenarios [21-22].

Nanoparticle-Reinforced Materials

Nanoparticles have shown great potential in pedodontic endodontics owing to their unique surface properties and multi-functionality. Because they have a high surface area/volume ratio, solubility is enhanced and biologic tissue interaction is promoted, enhancing drug efficacy through controlled release and enhanced bioavailability [23-24]. In bone regeneration, ternary nanocomplexes (TNCs) have shown the capacity to enhance maturation of the cells of the bone, reflecting promise in applications of tissue regeneration. Nanoparticle-containing materials are also multifunctional, allowing for the combination of targeting, imaging, and therapeutic functions [23]. Nanoparticle-loaded sealers in pediatric endodontics have shown 40% higher antibacterial activity against Enterococcus faecalis than regular sealers, which helps improve disinfection results [25]. Nanoparticles also penetrate more deeply into dentinal tubules, around 500 μm, further boosting efficacy in pediatric patients [25]. A variety of nanoparticles, such as silver, chitosan, and hydroxyapatite, have demonstrated great potential, with flexibility for tailoring treatment options in clinical applications [26]. Their combined antibacterial, bioactive, and regenerative properties render them beneficial in pediatric endodontic treatment [25-26].

The various uses and properties of the different materials and the related citations are summarized in Table 2.

**Table 2:** Types of Bioactive Materials With Their Uses MTA: mineral trioxide aggregate, CEM: calcium-enriched mixture.

Material	Key Uses and Properties	Citation
MTA	High sealing ability, biocompatible, stimulates hydroxyapatite growth. Used for vital pulp therapy, perforation repair, apexification, root-end filling, and regeneration. Limitations: long setting time, costly, risk of discoloration.	[11-12]
Biodentine	Calcium silicate-based, faster setting than MTA, less risk of discoloration, easy handling. Used for pulp capping, pulpotomy, apexification, perforation repair, and retrograde filling. Promotes dentin regeneration.	[13-15]
CEM Cement	Comparable to MTA in pulpotomy and apexogenesis. Good biocompatibility, osseoconductivity, and short setting time promote dentinal bridges. Used for pulp therapy, apexogenesis, and resorption repair.	[16-19]
Bioceramic Sealers	Excellent sealing, strong dentin adhesion, highly biocompatible, supports bone healing, used in primary and retreatment endodontics. High clinical success rates.	[20-22]
Nanoparticle-Reinforced Materials	Enhanced antibacterial action, better tissue penetration (500 nm), supports regeneration, and controlled drug release. Used in sealers and scaffolds for improved disinfection and healing.	[23-26]

Emerging and experimental bioactive materials

Graphene-Based Materials

Graphene-based materials (GBMs) have been found to possess significant potential to improve treatment outcomes in pediatric endodontics because of their unique properties. Graphene oxide (GO) is known to have strong antibacterial properties against cariogenic and periodontal pathogens, which is essential in the prevention of infection in root canal systems [27]. GO coatings on Ni-Ti pediatric rotary files through electrophoretic deposition (EPD) create uniform and continuous layers, which can potentially enhance mechanical strength and durability [28]. Moreover, GO-based composites are bioactive and induce mineral deposition, facilitating pulp healing and demonstrating promise in regenerative treatment. GO-CaF₂ and GO-Ag-CaF₂ composites have also shown efficient sealing on dentin surfaces. Further, the biocompatibility of these materials is dependent on particle shape, size, and concentration, all of which are critical parameters for safe dental application. These features altogether highlight GBMs as a promising agent for improving pediatric endodontic treatment [27-28].

Hydrogel-Based Scaffolds

Hydrogel scaffolds have been discovered to have significant potential in pediatric endodontics, particularly in regenerative endodontic therapies (REPs), as they are capable of aiding dentin-pulp complex regeneration through localized growth factor and drug delivery [29-30]. Hydrogel scaffolds are extremely biocompatible by mimicking the extracellular matrix of dental pulp [29] and can be easily loaded with bioactive molecules for long-term local release [29-30]. Hydrogels at 150-250 μg/mL Benzyldimethyldodecylammonium Chloride (BDMDAC) had robust antimicrobial efficacy against five bacterial pathogens [30] with more than 70% viability of dental pulp stem cells [30]. Porcine extracellular matrix (P-ECM) hydrogels also effectively deliver and release critical growth factors like bone morphogenetic protein-2 (BMP-2), transforming growth factor beta-1 (TGF-β1), basic fibroblast growth factor (bFGF), and vascular endothelial growth factor (VEGF), which aid tissue regeneration. Taken together, all these features emphasize hydrogel-based scaffolds as a worthy tool for improving regenerative outcomes in pediatric endodontics [29-30].

Bioactive Glass

Bioactive glass (BG) has been highly promising in pediatric endodontics because of its remineralizing nature and regenerative ability in dentin. By initiating hard tissue repair via biocompatibility and forming hydroxyapatite crystals, BG mimics the tooth structure naturally [31-32]. BG cement with LDN-193189 has been shown to have the potential to induce dental pulp cell differentiation into odontoblasts and dentin-like crystal formation. Regarding sealing efficiency, BG-based sealers are as effective as bioceramic sealers and lack cytotoxicity, indicating high biocompatibility [32]. Moreover, the chemical similarity of BG to human bone and dentin lies primarily in its ability to form hydroxyapatite upon contact with tissue fluids, closely resembling the mineral phase of natural hard tissues, though not necessarily in identical proportions [31-32]. These properties indicate that BG can enhance the outcomes of pediatric endodontics and reduce the risks of complications such as microleakage, bacterial reinfection, and adverse inflammatory responses, thereby improving treatment predictability [31-32].

Stem Cell-Compatible Scaffolds

Stem cell-compatible scaffolds have demonstrated potential in pediatric endodontics, especially for regenerative treatment of immature permanent teeth with necrotic pulps [33]. Puramatrix™ (Corning Inc., Corning, USA), a self-assembling peptide hydrogel, facilitated dental pulp stem cell (DPSC) growth and differentiation, with cells living for a minimum of three weeks and expressing odontoblastic markers dentin matrix protein-1 (DMP-1) and dentin sialophosphoprotein (DSPP) after 21 days [34]. In addition, 3D-printed gelatin scaffolds with BDMDAC exhibited antimicrobial effects against endodontic bacteria, with more than 70% DPSC viability and a six-month stability period. Platelet-poor plasma scaffolds loaded with umbilical cord mesenchymal stem cells exhibited therapeutic efficacy in mature teeth with apical periodontitis and root perforation. These scaffolds are promising materials for pediatric regenerative endodontic treatments [33-34]. 

Applications in pediatric endodontic procedures

Biomaterials have greatly contributed to pediatric endodontic treatments by increasing pulp vitality maintenance and promoting tissue healing. In direct and indirect pulp capping, biomaterials such as Biodentine and MTA are the most used, with Biodentine registering great success in pulp vitality maintenance of permanent immature teeth and carious exposures [35]. For pulpotomies, in which the coronal pulp is removed but radicular pulp is retained, healing and root development are facilitated by bioactive materials. Zinc bioactive glass achieved 94.74% clinical and 92.11% radiographic success at 12 months [36], while Biodentine presented similar results to formocresol within the same time frame [35]. The materials have advantages in handling, setting time, and dentinal bridge formation and are effective substitutes for conventional agents in pediatric endodontics [36].

Biomaterials are crucial in pediatric endodontics, particularly in apexogenesis and apexification. Apexogenesis targets the maintenance of root development in immature vital teeth through materials like CEM cement, MTA, and hydrogel-based scaffolds. Apexification for root-end closure of non-vital teeth frequently employs MTA as an apical barrier and bioceramic materials for thorough obturation. Research suggests that full obturation with MTA or Biodentine can provide an effective alternative to conventional methods and, theoretically, support tooth structure. However, further clinical trials are necessary to compare long-term results [37-38]. Material selection in pediatric endodontics should take into account patient age, tooth maturity, and behavioral factors [30-31]. Younger children, who are less likely to cooperate during lengthy procedures, benefit from faster-setting materials with reduced chair time, such as Biodentine or CEM cement. Immature permanent teeth require materials that promote continued root development, such as MTA and calcium-enriched cements [32]. In addition, the choice of material may depend on the need for easy handling and minimal postoperative discomfort, which is especially important among children with dental anxiety or special healthcare needs. These practical aspects should be weighed alongside biological properties to optimize outcomes for pediatric patients [34].

To complement these clinical applications, Table 3 summarizes the key components and material classes of the main bioactive agents, highlighting how their composition supports their biological and functional properties in pediatric endodontics.

**Table 3:** Composition of Bioactive Materials Used in Pediatric Endodontics MTA: mineral trioxide aggregate, CEM: calcium-enriched mixture.

Material	Material Class	Key Components	Functional Additives	Citation
MTA	Calcium silicate-based bioceramic	Tricalcium silicate, dicalcium silicate, bismuth oxide (radiopacifier), calcium aluminate	Promotes hydroxyapatite formation	[11-12]
Biodentine	Calcium silicate-based	Tricalcium silicate, zirconium oxide (radiopacifier), calcium carbonate	Faster setting, less discoloration than MTA	[13-15]
CEM Cement	Calcium-enriched bioceramic	Calcium oxide, calcium phosphate, calcium silicate, sulfur trioxide	Promotes dentin bridge, short setting time	[16-19]
TheraCal LC (Bisco Inc., Schaumburg, USA)	Resin-modified calcium silicate	Portland cement (calcium silicates), resin matrix, barium glass	Light-cured, immediate setting, fluoride release	[20-21]
EndoSequence BC Sealer	Premixed bioceramic	Calcium silicates, zirconium oxide, calcium phosphate monobasic	Injectable, ready-to-use, excellent sealing	[20-21]
ACTIVA BioACTIVE (Pulpdent Corporation, Watertown, USA)	Resin-modified glass ionomer hybrid	Bioactive ionic resin, rubberized resin, glass fillers	Releases calcium, phosphate, fluoride; shock absorption	[20-21]

Biological advantages and clinical superiority

With their biocompatibility and clinical efficacy, bioactive materials have exhibited considerable merits in pediatric endodontics. Calcium silicate-based materials like Biodentine induce dentinogenesis by precipitating reparative dentin formation, thus being particularly valuable in open-appearing cases [4]. The materials possess highly desirable physicochemical qualities and biocompatibility, favoring their long-term application among children [4]. In addition, bioactive bioceramic endodontic materials induce healing of the pulpal and periapical tissues, leading to better clinical success [3]. Their ease of handling is especially beneficial in pediatric dentistry, by making it easier to use and apply materials when there is poor patient cooperation [3]. In addition, the bioactivity of bioactive materials makes it possible to use them in multiple procedures, like pulp capping and pulpotomy. They can also act as retrograde filling materials, making them versatile for use in a wide range of different clinical situations. These coexisting attributes render bioactive materials extremely well-suited to contribute to the success of endodontic treatment in pediatric dentistry [3-4].

Limitations and practical concerns

Despite the advantages of using bioactive materials in pediatric endodontics, several issues need to be addressed. High costs and limited availability can make it hard to use them in some clinical settings where resources are tight. Difficulties in handling and long setting times with MTA may be challenging during procedures for young children who might not sit still for long treatments [39]. Also, the risk of tooth staining with MTA raises concerns about the appearance of the front teeth. Another major issue is the lack of long-term clinical data, making it challenging to evaluate the effectiveness and longevity of these materials in younger patients [39]. On top of that, some biosilicate cements behave differently from regular materials, forcing dentists to change their usual clinical methods [39]. These factors highlight the need for careful material selection and further research to improve the use of bioactive materials in pediatric endodontic care [39]. Beyond cost and handling, the use of bioactive materials is also influenced by regulatory approvals, regional availability, and healthcare infrastructure. For example, some materials may be widely adopted and approved in Europe or North America but less accessible in low- and middle-income countries due to higher costs or limited distribution [35-36]. Regulatory agencies in different countries may also approve materials for specific indications only, which can affect clinical choices. Furthermore, the uptake of newer materials in public healthcare settings may be delayed due to budget constraints or a lack of training. It is essential to take into account these practical and regulatory factors when implementing research findings into real-world pediatric dental care across different regions [38-39].

Current research trends and future directions

Existing research trends in pediatric endodontics with bioactive materials revolve around new ways of pulp regeneration and tissue repair. The development of intelligent biomaterials that react to physiological as well as environmental cues is also being explored to enable targeted healing in endodontic treatment [5,7]. Multifunctional scaffolds are also being explored for their potential to integrate enhanced biocompatibility, antibacterial properties, and mechanical properties, providing comprehensive support for regenerative treatments. While not explicitly outlined, analysis of over 2,500 articles shows an increasing focus on 3D printing and biofabrication methods to create accurately engineered scaffolds for dental use [5]. Emerging directions include the design of new hydraulic materials and the inclusion of nanomaterials to address current issues related to material handling, setting time, and durability over long periods of time. On the other hand, newer formulations like Five Mineral Oxides (5MO) and MTA Repair HP (Angelus, Londrina, Brazil) remain at the experimental stage: ex vivo and in vitro studies suggest strong biofilm suppression, biocompatibility, and favorable elemental composition for regeneration, but they lack pediatric clinical trial evidence to date [44]. Together, these developments seek to maximize the function of bioactive materials by enhancing their sealing ability, biological compatibility, and ability to aid in functional tissue regeneration for pediatric endodontic treatment, thus delivering better clinical success and long-term outcomes [5,7].

## Conclusions

Bioactive materials are increasingly acknowledged for their significant role in supporting pediatric endodontic practices that emphasize biological preservation and long-term dental health. As the field continues to advance, these materials provide promising pathways for the development of minimally invasive techniques specifically designed to address the unique needs of growing patients. Ongoing research and innovation are anticipated to further enhance their clinical applications, particularly through advancements in regenerative and personalised treatment strategies. Nevertheless, their use is not without limitations, as challenges such as higher costs, handling difficulties, risk of tooth discoloration, and limited long-term clinical data remain considerations for practitioners. Ultimately, bioactive materials contribute to treatment methodologies that align with the objectives of preserving natural dentition and fostering optimal oral development in children. Clinicians are encouraged to balance the current evidence with material availability, child‑specific needs, and practical considerations when selecting bioactive materials for treatment.

## References

[1] Şahin TN, Çakır A (2023). Deciphering pediatric root canal practices in turkey: a comparative study bridging the gap between practice and literature. Med Sci Monit.

[2] Camp JH (1991). Overviews of pediatric-endodontics. Alpha Omegan.

[3] Walsh RM, He J, Schweitzer J, Opperman LA, Woodmansey KF (2018). Bioactive endodontic materials for everyday use: a review. Gen Dent.

[4] Nadgouda M, Patel A, Chandak M, Sedani S, Sarangi S (2024). The management of open apex using a bioactive material: a case report. Cureus.

[5] Zhang L, Chen Z (2022). Bioactive materials in endodontics (Article in Simplified Chinese). Zhonghua Kou Qiang Yi Xue Za Zhi.

[6] Leveque M, Bekhouche M, Farges JC, Aussel A, Sy K, Richert R, Ducret M (2023). Bioactive endodontic hydrogels: from parameters to personalized medicine. Int J Mol Sci.

[7] Zafar K, Jamal S, Ghafoor R (2020). Bio-active cements-mineral trioxide aggregate based calcium silicate materials: a narrative review. J Pak Med Assoc.

[8] Primus CM, Tay FR, Niu LN (2019). Bioactive tri/dicalcium silicate cements for treatment of pulpal and periapical tissues. Acta Biomater.

[9] Keane TJ, Badylak SF (2014). Biomaterials for tissue engineering applications. Semin Pediatr Surg.

[10] Afnan MA, Saxena AK (2018). Tissue repair in neonatal and paediatric surgery: analysis of infection in surgical implantation of synthetic resorbable biomaterials. Biomed Mater Eng.

[11] Parirokh M, Torabinejad M (2010). Mineral trioxide aggregate: a comprehensive literature review--part III: clinical applications, drawbacks, and mechanism of action. J Endod.

[12] Torabinejad M, Parirokh M, Dummer PM (2018). Mineral trioxide aggregate and other bioactive endodontic cements: an updated overview - part II: other clinical applications and complications. Int Endod J.

[13] Malkondu Ö, Karapinar Kazandağ M, Kazazoğlu E (2014). A review on biodentine, a contemporary dentine replacement and repair material. Biomed Res Int.

[14] Rajasekharan S, Martens LC, Cauwels RG, Verbeeck RM (2014). Biodentine™ material characteristics and clinical applications: a review of the literature. Eur Arch Paediatr Dent.

[15] Rajasekharan S, Martens LC, Cauwels RG, Anthonappa RP (2018). Biodentine™ material characteristics and clinical applications: a 3 year literature review and update. Eur Arch Paediatr Dent.

[16] Mehrdad L, Malekafzali B, Shekarchi F, Safi Y, Asgary S (2013). Histological and CBCT evaluation of a pulpotomised primary molar using calcium enriched mixture cement. Eur Arch Paediatr Dent.

[17] Nosrat A, Seifi A, Asgary S (2013). Pulpotomy in caries-exposed immature permanent molars using calcium-enriched mixture cement or mineral trioxide aggregate: a randomized clinical trial. Int J Paediatr Dent.

[18] Asgary S, Aram M, Fazlyab M (2024). Comprehensive review of composition, properties, clinical applications, and future perspectives of calcium-enriched mixture (CEM) cement: a systematic analysis. Biomed Eng Online.

[19] Utneja S, Nawal RR, Talwar S, Verma M (2015). Current perspectives of bio-ceramic technology in endodontics: calcium enriched mixture cement - review of its composition, properties and applications. Restor Dent Endod.

[20] Al-Haddad A, Che Ab Aziz ZA (2016). Bioceramic-based root canal sealers: a review. Int J Biomater.

[21] Giacomino CM, Wealleans JA, Kuhn N, Diogenes A (2019). Comparative biocompatibility and osteogenic potential of two bioceramic sealers. J Endod.

[22] Haji TH, Selivany BJ, Suliman AA (2022). Sealing ability in vitro study and biocompatibility in vivo animal study of different bioceramic based sealers. Clin Exp Dent Res.

[23] Szelenyi I (2012). Nanomedicine: evolutionary and revolutionary developments in the treatment of certain inflammatory diseases. Inflamm Res.

[24] Singh L, Kruger HG, Maguire GE, Govender T, Parboosing R (2017). The role of nanotechnology in the treatment of viral infections. Ther Adv Infect Dis.

[25] Kader AA, Chandran S, Unnikrishnan KK, Mannur NA, Gupta A, Subramani SK (2024). Investigation of the potential of nanoparticles as a new drug delivery system for endodontic treatment. J Pharm Bioallied Sci.

[26] Raura N, Garg A, Arora A, Roma M (2020). Nanoparticle technology and its implications in endodontics: a review. Biomater Res.

[27] Nizami MZ, Yin IX, Lung CY, Niu JY, Mei ML, Chu CH (2022). In vitro studies of graphene for management of dental caries and periodontal disease: a concise review. Pharmaceutics.

[28] Panja K, A VS, N V, Ramar K (2024). Surface coating of nickel-titanium (Ni-Ti) pediatric rotary file using graphene oxide: a scanning electron microscopy analysis. Cureus.

[29] Abbass MM, El-Rashidy AA, Sadek KM (2020). Hydrogels and dentin-pulp complex regeneration: from the benchtop to clinical translation. Polymers (Basel).

[30] Dallos Ortega M, Aveyard J, Magdy Abdelgawad R (2025). Antimicrobial 3D printed gelatin scaffolds for root canal disinfection in regenerative endodontics procedures. Biomater Sci.

[31] Profeta AC, Prucher GM (2017). Bioactive-glass in endodontic therapy and associated microsurgery. Open Dent J.

[32] Huang G, Liu SY, Wu JL, Qiu D, Dong YM (2022). A novel bioactive glass-based root canal sealer in endodontics. J Dent Sci.

[33] Raddall G, Mello I, Leung BM (2019). Biomaterials and scaffold design strategies for regenerative endodontic therapy. Front Bioeng Biotechnol.

[34] Cavalcanti BN, Zeitlin BD, Nör JE (2013). A hydrogel scaffold that maintains viability and supports differentiation of dental pulp stem cells. Dent Mater.

[35] Brizuela M, Daley JO (2025). Dental materials: Biodentine, a calcium silicate bioactive. https://www.ncbi.nlm.nih.gov/books/NBK599506/.

[36] Reddy VN, Reddy D, Snehitha K, Narahari S, Prasad PS, Rehaman T (2025). Clinical radiographic and histological evaluation of zinc bioactive glass as a pulpotomy medicament in primary molar teeth: an in vivo study. Int J Clin Pediatr Dent.

[37] Rao MH, Krishnan R, Kumaraswamy M, Keshav A, Gopal P, Thomas E (2024). Assessment of treatment outcomes with complete orthograde obturation with bioceramic materials: a scoping review. J Contemp Dent Pract.

[38] Darak P, Likhitkar M, Goenka S, Kumar A, Madale P, Kelode A (2020). Comparative evaluation of fracture resistance of simulated immature teeth and its effect on single visit apexification versus complete obturation using MTA and biodentine. J Family Med Prim Care.

[39] Acharya S, Raghunath N, Mallikarjun RM, Nalawade T, Gurunathan D, Godhi BS (2024). Bioactive biosilicate cements in pediatric dentistry - a review of the latest materials. J Pharm Bioallied Sci.

[40] Uyar DS, Alacam A (2021). Evaluation of partial pulpotomy treatment in cariously exposed immature permanent molars: randomized controlled trial. Niger J Clin Pract.

[41] Gopinath VK, Pulikkotil SJ, Veettil SK, Dharmarajan L, Prakash PS, Dhar V, Jayaraman J (2022). Comparing the clinical and radiographic outcomes of pulpotomies in primary molars using bioactive endodontic materials and ferric sulfate - a systematic review and meta-analysis of randomized clinical trials. J Evid Based Dent Pract.

[42] Abdellatif D, Iandolo A, De Benedetto G, Giordano F, Mancino D, Euvrard E, Pisano M (2024). Pulp regeneration treatment using different bioactive materials in permanent teeth of pediatric subjects. J Conserv Dent Endod.

[43] Alsanouni M, Bawazir OA (2019). A randomized clinical trial of NeoMTA plus in primary molar pulpotomies. Pediatr Dent.

[44] Abu Hasna A, de Paula Ramos L, Campos TM, de Castro Lopes SL, Rachi MA, de Oliveira LD, Carvalho CA (2022). Biological and chemical properties of five mineral oxides and of mineral trioxide aggregate repair high plasticity: an in vitro study. Sci Rep.

